# Long term outcome of surgical treatment of chondroblastoma: analysis of local control and growth plate/articular cartilage related complications

**DOI:** 10.1186/s12891-023-06239-7

**Published:** 2023-02-22

**Authors:** Francesco Muratori, Roberto Scanferla, Giuliana Roselli, Filippo Frenos, Domenico Andrea Campanacci

**Affiliations:** 1 Department of Orthopaedic Oncology and Reconstructive Surgery, AOU Careggi, Florence, Italy; 2 Department of Radiology, AOU Careggi, Florence, Italy

**Keywords:** Chondroblastoma, Epiphysis, Aggressive curettage, Recurrence, Osteoarthritis

## Abstract

**Background:**

Chondroblastoma (CBL) is a rare benign chondroid producing bone tumor that typically occurs in epiphysis or apophysis of growing children and young adults. Intralesional curettage is the treatment of choice, while resection is required in selected cases, even though the use of minimally invasive ablation techniques has been advocated. Authors reviewed a series of 75 CBLs with the aim of assess risk factors for local recurrence, the growth plate related complications after epiphyseal curettage and the risk of arthritis of the adjacent joint after epiphyseal curettage.

**Methods:**

We retrospectively review 69 CBLs treated with intralesional curettage and 6 treated with resection from March 1995 to February 2020. The median age was 18.8 years (7 to 42, median 16). The site was proximal humerus in 18 cases, proximal tibia in 17, distal femur in 16, talus in 6, femur’s head in 4, calcaneus in 3, acromion in 3, trochanteric region in 2, distal tibia in 2, patella in 2, supracetabular region in 1 and distal humerus in 1 patient.

**Results:**

Mean follow-up was 124.2 months (24 to 322, median 116). Among patients treated with curettage, 7.3% of local recurrence was observed and 12 (17.4%) patients developed osteoarthritis of the adjacent joint. Five patients (7.3%) presented limb length discrepancy of the operated limb ranging from 0.5 to 2 cm. Recurrence free survival rate was 94.2% at 5 and 91.6% at 10 years. A mean Musculoskeletal Tumor Society (MSTS) of 29.3 points (20 to 30, median 30) was observed.

**Conclusion:**

More than 90% of CBLs were successfully treated with aggressive curettage but segmental resection is required in selected cases. In a relatively small proportion of cases long term complications can occur due to growth plate damage or osteoarthritis.

**Trial Registration:**

Retrospectively registered.

## Background

Chondroblastoma (CBL) is a rare benign locally aggressive tumor of bone typical of children and young adults with predilection for epiphyseal or apophyseal regions, composed of chondroblastic cells and islands of eosinophilic chondroid matrix [1], that most frequently occurs in proximal tibia, proximal femur and proximal humerus [2–4]. It is classified as benign tumor although lung metastasis have been reported [3, 5].

Clinical presentation includes pain, swelling, local tenderness and reduced range of motion of the joint [2, 4]. Although the clinical features and radiological images are quite typical, differential diagnosis with giant cell tumor of bone, aneurismal bone cyst or other benign bone lesion is necessary. In some cases, an aggressive presentation at imaging, with intense reactive inflammatory signs, may also simulate malignancy. In recent studies, a K36M mutation in the H3F3A or H3F3B genes was identified, and it was found to be specific in 70% to 95% of CBL [6]. At the same time, a specific G34M mutation in H3F3A was found in giant cell tumor of bone, supporting differential diagnosis [6].

Surgery represents the mainstay of treatment, with aggressive curettage of the tumor followed by filling with bone grafts or cement recommended in most cases [3, 4, 7]. Curettage of the epiphyseal lesion can result in a damage of the growth plate or articular cartilage leading to reduced joint function, limb length discrepancy or deformity [7]. Growth disorders and growth plate injuries related to tumor destruction and aggressive curettage with local adjuvants such as liquid nitrogen or phenol in patients with open physis are reported in literature [2, 8–10]. Moreover, secondary osteoarthritis after extensive epiphyseal curettage has been described [2, 11, 12].

Partial or total resection of the epiphysis is advocated in aggressive lesions, with articular cartilage destruction in which the tumoral extension does not allow a joint-sparing procedure. In these cases, osteoarticular allograft, allograft-prosthesis composites (APC), conventional and modular prostheses have been used for reconstruction [3, 4]. Another treatment option reported for small lesions localized in challenging surgical sites as femoral head, posterior aspect of proximal tibia and tarsal bones is radiofrequency ablation (RFA) [3].

Local recurrence (LR) rate after treatment varies considerably, ranging from 0 to 39% [2–4, 7, 8, 12–16]. No significative risk factors for recurrence were identified, even though epiphyseal site, young age and secondary aneurismal bone cyst (ABC) component at time of diagnosis were associated to an increased risk [2–4, 12, 17, 18].

The purpose of our study was to review retrospectively a series of 75 patients, surgically treated for CBL, analyzing type of treatment, incidence of recurrence, complications and functional results, with the aim to answer the following questions: 1) what are the risk factors for local recurrence of CBL? 2) what are growth plate related complications after epiphyseal curettage? 3) is there an increased risk of arthritis of the adjacent joint after epiphyseal curettage and does the site influence this long-term complication?

## Methods

We retrospectively reviewed a series of 75 patients treated for CBL in our Unit from March 1995 to February 2020, analyzing several parameters such as age, gender, anatomical site, stage, proportion of subchondral bone involvement, presence of pathological fracture, surgical treatment (curettage/resection), reconstruction, type of filling after curettage, complications, recurrence, incidence of metastasis and functional results. Fifty-four patients were male (72%) and twenty-one were female (28%). Patients undergoing open surgical treatment were included, while patients treated with mini-invasive technique were excluded. The mean age of patients at the time of diagnosis was 18.8 years (7 to 42, median 16) and the mean follow-up was 124.2 months (24 to 322, median 116). Pain was the most frequent symptom, associated with local swelling and joint effusion with functional limitation. No pathological fracture at the diagnosis was observed.

The site of CBL was proximal humerus in 18 cases (24%), proximal tibia in 17 (22.7%), distal femur in 16 (21.3%), talus in 6 (8%), femur’s head in 4 (5.3%), calcaneus in 3 (4%), acromion in 3 (4%), trochanteric region in 2 (2.7%), distal tibia in 2 (2.7%), patella in 2 (2.7%), supracetabular region in 1 (1.3%) and distal humerus in 1 (1.3%) (Table 1).

**Table 1:** Patients’ characteristics

**Characteristics**	Number
Patients	75
*Males*	*54 (72%)*
*Females*	*21 (28%)*
Age	*18.8 (7 – 42)*
Site	
*Proximal humerus*	*18 (24%)*
*Proximal tibia*	*17 (22.7%)*
*Distal femur*	*16 (21.3%)*
*Talus*	*6 (8%)*
*Femur’s head*	*4 (5.3%)*
*Calcaneus*	*3 (4%)*
*Acromion*	*3 (4%)*
*Trochanteric region*	*2 (2.7%)*
*Distal tibia*	*2 (2.7%)*
*Patella*	*2 (2.7%)*
*Supracetabular region*	*1 (1.3%)*
*Distal humerus*	*1 (1.3%)*
Secondary ABC component	21 (28%)
*Distal femur*	*5 (23.8%)*
*Proximal tibia*	*4 (19.1%)*
*Proximal humerus*	*3 (14.3%)*
*Talus*	*3 (14.3%)*
*Calcaneus*	*3 (14.3%)*
*Proximal femur*	*2 (9.6%)*
*Patella*	*1 (4.9%)*

At surgery, in patients treated with intralesional curettage, the physis was open in 21 patients (30.0%), closing in 13 (19%) and closed in 35 (51%) (Table 2). The physis was considered as “open” if a clearly defined epiphyseal plate was evident, as “closing” if a thin and irregular epiphyseal plate was perceptible and as “closed” if an epiphyseal scar was present. According to Enneking staging system [19], at diagnosis tumor was radiologically latent in 17 cases (22.7%), active in 34 (45.3%) and aggressive in 24 (32%). Tumor stage was assessed preoperatively using CT and MRI exams. At the CT scan subchondral bone of the articular surface was involved with an extension of 20% in 8 patients, 30% in 4, 50% in 4 and 100% in one, while it was intact in all the others.

**Table 2:** Clinical features and treatment

Curettage	69 (92%)
Status of the Physis	
* Open*	*21 (30%)*
* Closing*	*13 (19%)*
* Closed*	*35 (51%)*
MSTS stage	
* Latent*	*17 (22.7%)*
* Active*	*34 (45.3%)*
* Aggressive*	*24 (32%)*
Local Adjuvants	
* High-speed Burr*	*64*
* Phenol*	*39*
* Hydrogen Peroxidase*	*24*
* Liquid Nitrogen*	*10*
* None*	*2*
Type of filling	
* Bone graft*	*61 (88.4%)*
+ *Demineralized Bone Matrix*	*5*
+ *Autologous bone marrow aspirate*	*2*
* Autologous bone graft*	*4 (5.8%)*
* Cement*	*2 (2.8%)*
* Osteoarticular graft*	*1 (1.5%)*
* None*	*1 (1.5%)*
**Resection**	**6 (8%)**

All cases of CBL had a confirmed histologic diagnosis: we performed an open biopsy in the first 10 patients for lack of available CT-machine, a CT-guided trocar biopsy in 22 and intra-operative frozen-sections before proceeding with curettage in 43. In 21 cases (28%), a secondary aneurismal bone cyst (ABC) component was observed at histological examination, 5 in distal femur, 4 in proximal tibia, 3 in proximal humerus, 3 in talus, 3 in calcaneus, 2 in proximal femur and 1 in patella (Table 1). Immunohistochemistry was performed to differentiate GCT (H3.3 G34W) and ABC while CBL/ABC ratio wasn’t available.

Regarding surgical treatment, curettage and filling of bone cavity was performed in 69 patients (92%) while resection was performed in 6 cases (8%). In patients treated with intralesional curettage, local adjuvants were used in different combination according to surgeon preference: liquid nitrogen was used in 10 cases, phenol in 39, hydrogen peroxidase in 24, high-speed burr was used in 64 cases, while in two patients no local adjuvants were used (Table 2). Local adjuvants were used alone or in association, according to tumoral size, location, growth plate and subchondral bone involvement. After curettage, the residual bone cavity was filled with allogenic bone graft in 61 patients, associated with DBM (Demineralized Bone Matrix) in 5 cases and with concentrated autologous bone marrow aspirate in 2, while autologous bone graft from iliac crest was used in 4 patients. Cement was used only in two cases and in one case, after curettage involving part of the articular surface of the proximal humerus, a reconstruction with an osteochondral allograft was performed. Finally, in one patient no filling was performed due to the small cavity dimension (Table 2). In 2 patients with proximal femur CBL involving trochanteric region, a preventive fixation with DHS (Dynamic Hip Screw) plate was performed.

**Fig. 1:**
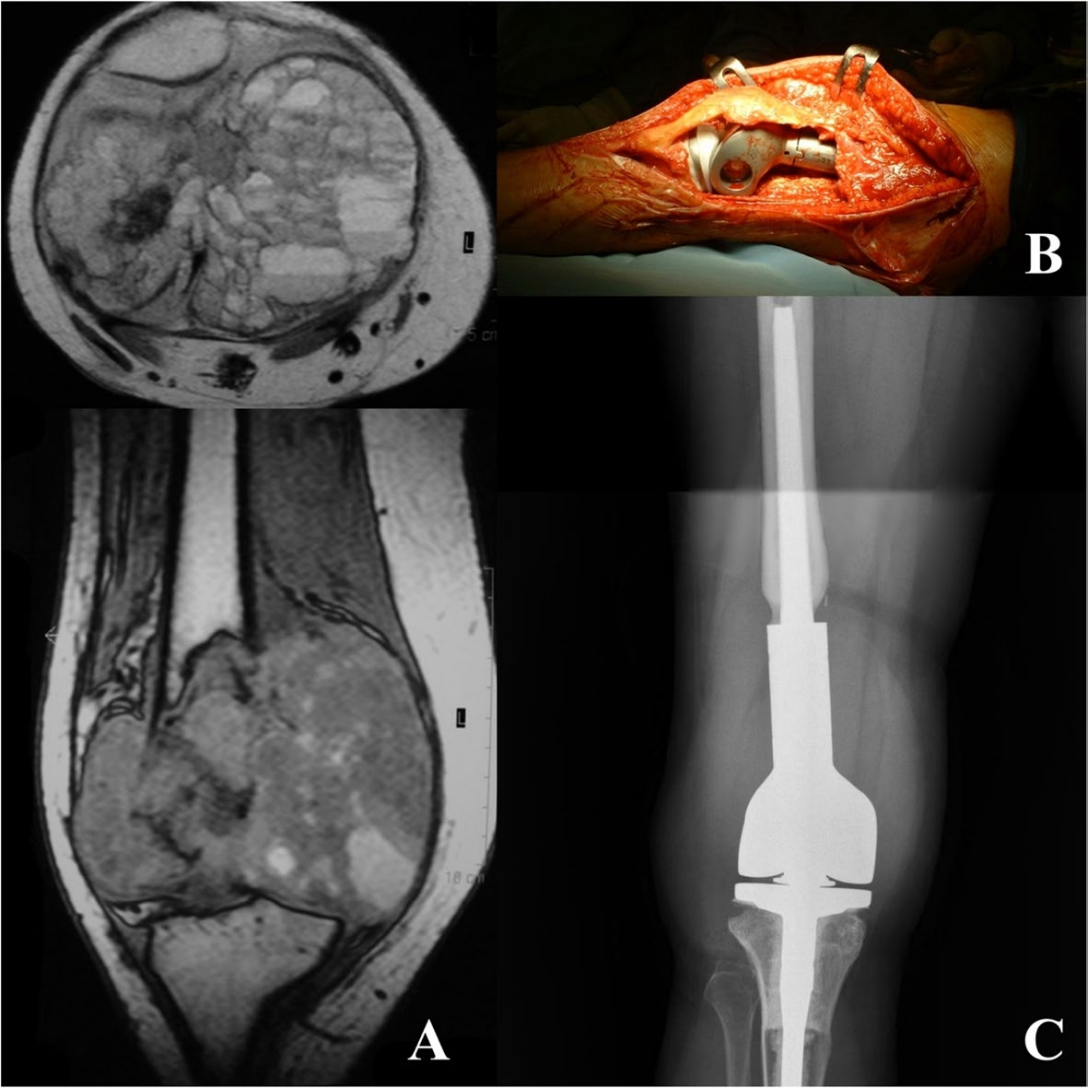
A 19 year-old patient with giant aggressive distal femoral CBL associated with ABC (**A**), treated with extraarticular knee resection and allograft-prosthesis composite reconstruction (**B**, **C**)

Six patients were treated with resection. One patient with proximal humeral aggressive lesion underwent to endoprosthetic reconstruction, another patient with femoral head aggressive lesion underwent to standard hemiarthroplasty and the last one CBL with large and complete involvement of the distal femoral epiphysis with invasion of joint, associated with ABC was treated with extraarticular knee resection and allograft-prosthesis composite reconstruction (Fig. 1). Three patients with acromion CBL received partial resection without reconstruction.

Functional outcome was assessed using Musculoskeletal Tumor Society (MSTS) score system [20] and Sailhan functional criteria [2].

### Statistical analysis

The statistical analysis was performed with version 16.8.4 of the MedCalc software. All variables were analyzed for their impact on local recurrence free survival with a 5- and 10-years follow-up. In the univariate analysis of general survival estimates, local recurrence-free survival was calculated using the Kaplan–Meier method. The values of *p* < 0.05 were considered statistically significant. The comparison of calculated survival curves was performed by the medium log-rank tests. Risk reports and confidence intervals (95%) were calculated using the Cox risk test. Significant parameters of univariate analyses have been incorporated into a Cox's multivariate regression model.

## Results

No early post-surgical complications were observed. At last clinical control no patient had lung metastasis detected with X-rays.

**Fig. 2:**
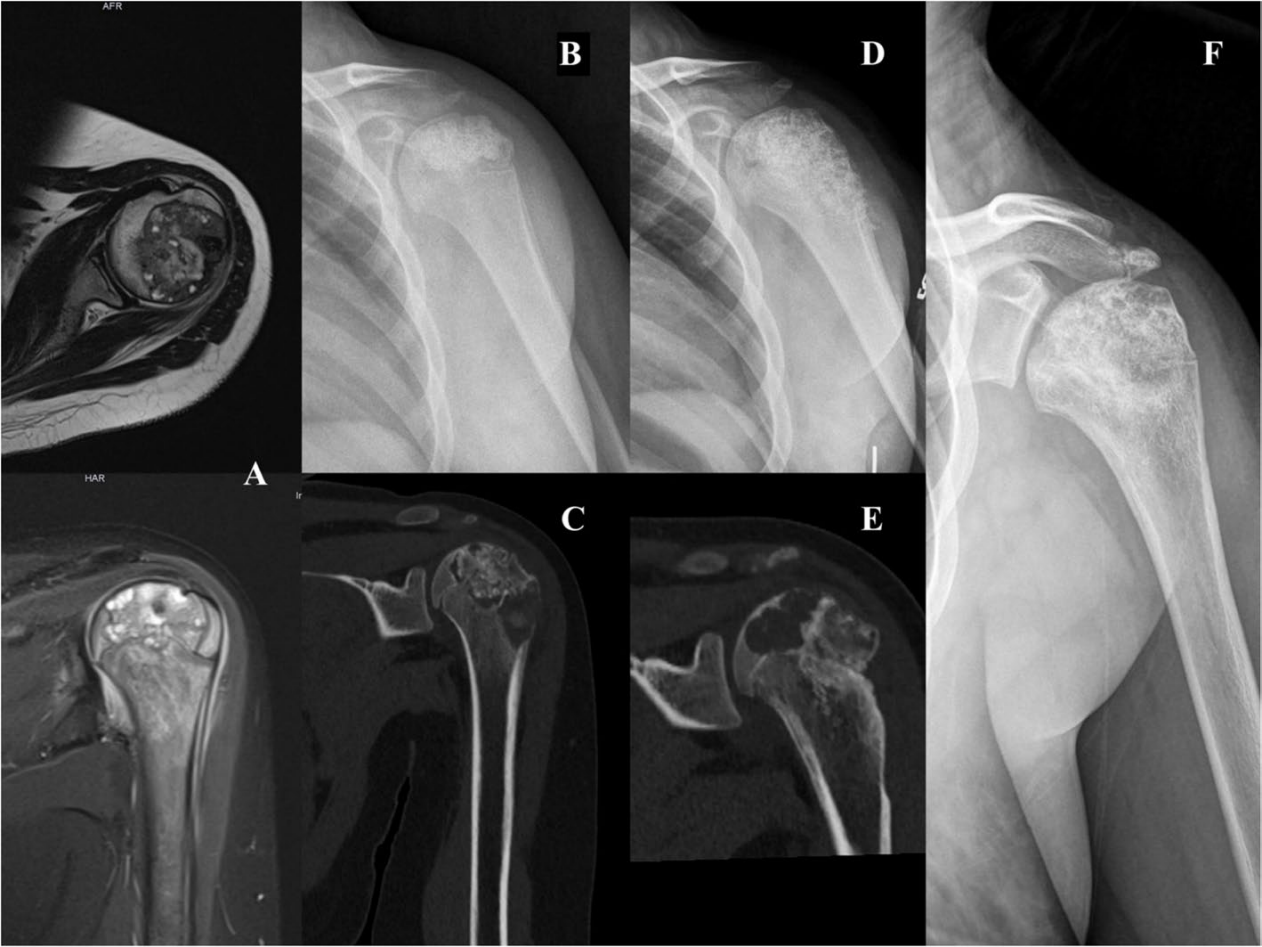
A 11-year-old girl with an active proximal humerus CBL with involvement of growth plate (**A**) developed two recurrences after 6 (**C**) and 12 months (**E**), both treated with a new aggressive curettage and filling with allogenic bone graft (**B**, **D**, **F**)

Considering patients treated with intralesional curettage, LR was observed in 5 cases (7.3%) after a mean of 30 months (5 to 111, median 11) (Table 3). A 11-year-old girl with talus CBL associated with ABC had 2 recurrences, the first 16 months after index surgery and the second 12 months after previous surgery; the first was treated with a new curettage and filling with allogenic bone grafting while the second was managed with RFA. A 14-year-old male had a recurrence of a CBL in the posterior aspect of proximal tibial epiphysis 7 months after curettage and cryotherapy; the recurrence was successfully treated with a new curettage. Another recurrence was observed after curettage of distal femur CBL 12 months after index surgery in a 40-year-old female; due to the extensive articular involvement the patient underwent distal femur resection and reconstruction with megaprosthesis (MegasystemC, Waldemar Link®, Germany). A 11-year-old girl with an active proximal humerus CBL with involvement of growth plate developed two recurrences, 6 months after index surgery and 6 months after second surgery respectively, both treated with a new aggressive curettage and filling with allogenic bone graft (Fig. 2). Lastly, a 27-year-old girl, after curettage of a distal femoral CBL with secondary ABC, developed a recurrence 111 months after index surgery, treated with a new curettage and bone grafting. In patients with recurrent lesion the mean age was 20.6 years (11 to 40, median 16). The physis was closed in three cases, closing in one and open in the last one; the lesion was radiographically inactive in one case, active in two and aggressive in the other two. Moreover, ABC component was evident in two patients (40%).

**Table 3:** Local recurrences, in relation to histology, stage according Enneking system, status of the physis and treatment. * time of second recurrence from previous surgery

Site	Histology	Stage	Physis	Treatment	Time LR (months)	Treatment LR	MSTS score
Talus	CBL + ABC	1	Closed	Curettage	16 12^*^	Curettage RFA	30
Acromion	CBL	3	Closed	Resection	128	Resection	30
Proximal tibia	CBL	2	Closing	Curettage	7	Curettage	30
Distal femur	CBL	3	Closed	Curettage	11	Resection	25
Proximal humerus	CBL	3	Open	Curettage	66^*^	Curettage Curettage	28
Distal femur	CBL + ABC	2	Closed	Curettage	111	Curettage	27

**Fig. 3:**
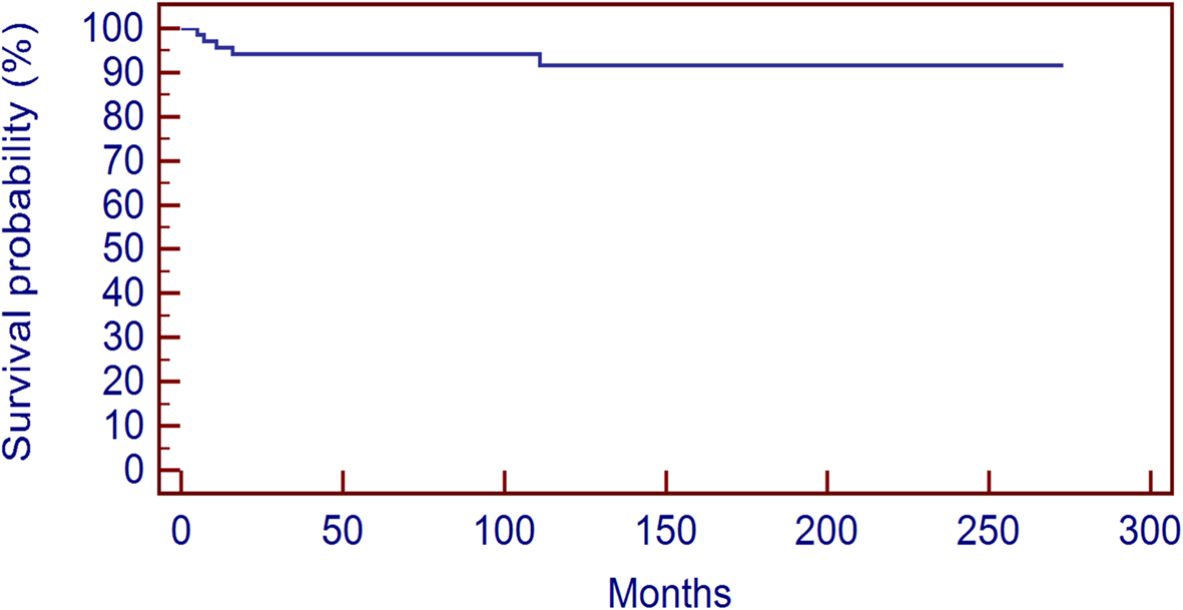
Recurrence free survival at 5 and 10 years

**Fig. 4:**
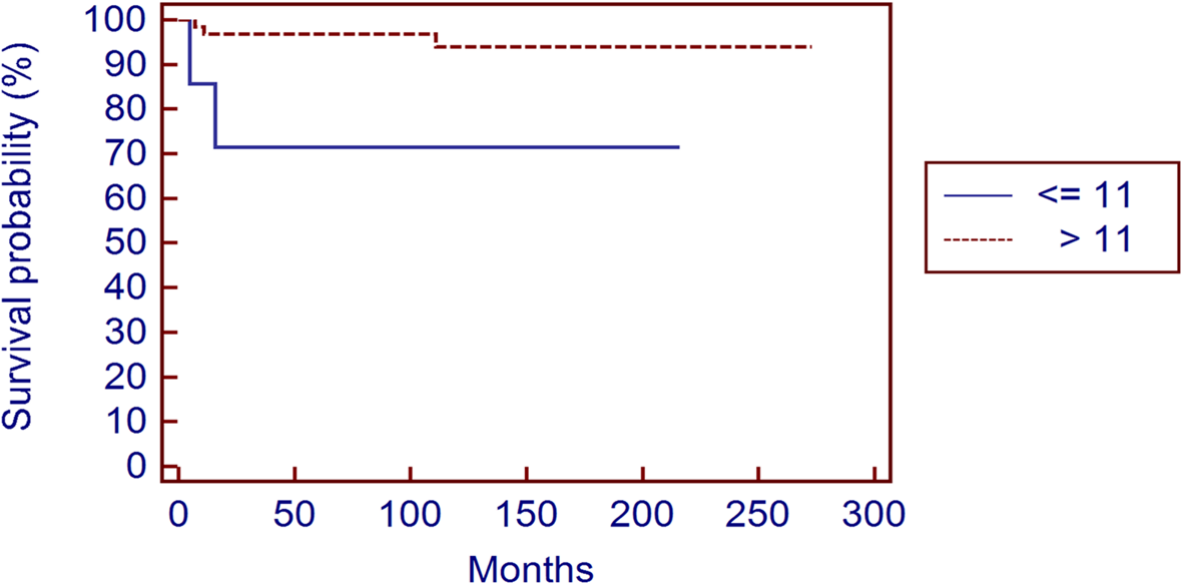
Recurrence free survival at 5 and 10 years according to age at surgery

One patient (16.6%) treated with resection for a CBL of the acromion developed a LR 128 months after primary surgery (Table 3). The mean time to local recurrence was of 46.3 months (5 to 128, median 13.5) and the local recurrence free survival (LRFS) rate was 94.2% at 5 and 91.6% at 10 years (Fig. 3). In our series the only significant factor that influenced local recurrence risk was the age at surgery < 11 years (*p* = 0.012) (Fig. 4); site (*p* = 0.776), stage (*p* = 0.687), status of the physis (*p* = 0.872), use of local adjuvants (*p* = 0.926), use of liquid nitrogen (*p* = 0.671) or ABC component at diagnosis (*p* = 0.514) didn’t statistically affect local recurrence risk (Table 4).

**Table 4:** Statistical analysis shows significant risk local adjuvants used (*p* 0.015), the use of liquid nitrogen (*p* 0.025) and the extension of subchondral bone involvement in lower limb (*p* 0.007); while stage (*p* 0.058), status of the physis (*p* 0.787) nor extension of subchondral bone involvement in upper limb (*p* 0.185) didn’t represent significant risk factors

Local recurrence		
Site	*Proximal humerus*	*p* 0.776
	*Proximal femur*	
	*Distal femur*	
	*Proximal tibia*	
	*Talus*	
	*Other sites*	
Stage	*Inactive*	*p* 0.687
	*Active*	
	*Aggressive*	
Age	> = *11*	***p* 0.012**
	> *11*	
Status of the physis	*Open*	*p* 0.872
	*Closing*	
	*Closed*	
Local adjuvants	*None*	*p* 0.926
	*One*	
	*Two or more*	
Liquid Nitrogen	*Yes*	*p* 0.671
	*No*	
ABC	*Yes*	*p* 0.514
	*No*	
**Osteoarthritis**		
Stage	*Inactive*	*p* 0.058
	*Active*	
	*Aggressive*	
Status of the physis	*Open*	*p* 0.787
	*Closing*	
	*Closed*	
Subchondral bone involvement in upper limb	*None*	*p* 0.185
	< *20%*	
	*20* > *50%*	
	> *50%*	
Subchondral bone involvement in lower limb	*None*	***p* 0.007**
	< *20%*	
	*20* > *50%*	
	> *50%*	
Local adjuvants	*None*	***p* 0.015**
	*One*	
	*Two or more*	
Liquid Nitrogen	*Yes*	***p* 0.025**
	*No*	

Long-term complication occurred in 8 patients (10.7%) (Table 5). At last follow-up, premature closure of the physis was observed in five patients (7.2%) with a CBL involving the growth plate, causing a limb length discrepancy of the affected limb ranging from 0.5 to 2 cm, in one case associated with a genu valgum. The site involved was proximal humerus, proximal and distal femur, proximal and distal tibia. Mean age of these patients was 12.6 (11 to 14, median 13). A 17 year-old boy with distal tibia CBL developed an Ewing’s Sarcoma in the same site 29 months after primary surgery. The patient underwent to pre- and post-operative chemotherapy and local treatment was performed with resection of distal tibia and reconstruction with ankle arthrodesis with autologous iliac crest and allogenic bone grafts. After 165 months follow-up, the patient was continuously disease free. A 11 year-old boy with an aggressive CBL of the right patella developed a chondropathy of the patella-femoral joint treated with multiple perforation. A 19-year-old patient treated with extraarticular resection of the knee due to an aggressive CBL had distal femoral prosthetic mechanical failure 188 months after primary surgery; he underwent to revision of the femoral component with excellent functional outcome at last clinical control.

**Table 5:** Growth plate and articular cartilage related complication

	**Growth plate related complication**		**Osteoarthritis**	
	**Yes**	No	**Yes**	No
**Site**				
Proximal humerus	1/17 (**5.8%**)	16/17 (94.2%)	2/17 (**11.8%**)	15/17 (88.2%)
Supracetabular region	0/1 (**0%**)	1/1 (100%)	1/1 (**100%**)	0/1 (100%)
Femur’s head	1/3 (**33.3%**)	2/3 (66.7%)	1/3 (**33.3%**)	2/3 (66.7%)
Distal femur	1/15 (**6.7%**)	14/15 (93.3%)	2/15 (**13.3%**)	13/15 (86.7%)
Patella	0/2 (**0%**)	2/2 (100%)	1/2 (**50%**)	1/2 (50%)
Proximal tibia	1/17 (**5.8%**)	16/17 (94.2%)	3/17 (**17.7%**)	14/17 (82.3%)
Distal tibia	1/2 (**50%**)	1/2 (50%)	0/2 (**0%**)	2/2 (100%)
Talus	0/6 (**0%**)	6/6 (100%)	2/6 (**33.3%**)	4/6 (66.7%)
**Stage**				
Latent	1/17 (**5.9%**)	16/17 (94.1%)	2/17 (**11.8%**)	15/17 (88.2%)
Active	1/34 (**2.9%**)	33/34 (97.1%)	4/34 (**11.8%**)	30/34 (88.2%)
Aggressive	3/18 (**16.7%**)	15/18 (83.3%)	6/18 (**33.3%**)	12/18 (66.7%)
**Physis status**				
Open	5/21 (**23.8%**)	16/21 (76.2%)	3/21 (**14.3%**)	18/31 (58.1%)
Closing	0/13 (**0%**)	13/13 (100%)	3/13 (**23.1%**)	10/13 (76.9%)
Closed	0/35 (**0%**)	35/35 (100%)	6/35 (**17.1%**)	29/35 (82.9%)
**Local adjuvants**				
High-speed burr	5/64 (**7.8%**)	59/64 (92.2%)	10/64 (**15.6%**)	54/64 (84.4%)
Phenol	3/39 (**76.9%**)	36/39 (92.3%)	1/39 (**2.6%**)	38/39 (97.4%)
Liquid Nitrogen	1/10 (**10%**)	9/10 (90%)	4/10 (**40%**)	6/10 (60%)
**Subchondral bone involvement**				
None	2/55 (**3.6%**)	53/55 (96.4%)	7/55 (**12.7%**)	48/55 (87.3%)
< 20%	2/6 (**33.3%**)	4/6 (66.7%)	2/6 (**33.3%**)	4/6 (66.7%)
20 > 50%	1/5 (**20%**)	4/5 (80%)	2/5 (**40%**)	3/5 (60%)
> 50%	0/3 (**0%**)	3/3 (100%)	1/3 (**33.3%**)	2/3 (66.7%)

As far as degenerative joint changes were concerned, at last follow up 12 patients (17.4%) treated with curettage developed osteoarthritis of the adjacent joint (Table 5). We observed degenerative changes in 3 proximal tibia CBL (3/17, 17.7%), in 2 proximal humerus (2/17, 11.8%), in 2 distal femurs (2/15, 13.3%), in one femoral head (1/3, 33.3%), in 2 tali (2/6, 33.3%), in one patella (1/2, 50%) and in one supracetabular region tumor (1/1, 100%). Eight patients were asymptomatic with radiographic signs of arthritis, three had mild symptoms without limitation of daily living activities, while one patient with CBL of the femoral head developed severe osteoarthritis, requiring total hip arthroplasty 13 years after primary surgery. Five of these patients had tumoral subchondral bone involvement ranging between 20% and more than 50%, while the others had intact articular surface. In four cases cryotherapy and in three cases phenol were used as local adjuvant. Four patients had a histologic diagnosis of secondary ABC. In our series significant risk factors for the articular degenerative changes were the amount of local adjuvants used (none vs one vs two or more, *p* = 0.015), the use of liquid nitrogen (*p* = 0.025) and the extension of subchondral bone involvement in lower limb (none vs < 20% vs 20 > 50% vs > 50%, *p* = 0.007), while stage (*p* = 0.058), status of the physis (*p* = 0.787) nor extension of subchondral bone involvement in upper limb (*p* = 0.185) didn’t represent significant risk factors (Table 4).

MSTS score system [19] was used for assessment of functional results. The mean score was 29.3 points (20 to 30, median 30). Sixty-nine patients treated with curettage had a mean MSTS score of 29.4 (23 to 30, median 30) while six patients treated with resection had a mean score of 27.4 points (20 to 30, median 28.5). Among 64 patients treated with curettage with no recurrence, 50 (78%) had unrestricted activities including sports and normal joint mobility, 11 (17%) had unrestricted activities with occasional pain, effusion or limb-length discrepancy and 3 (5%) had modification of their daily activities. Among 5 patients treated with curettage followed by local recurrence, 2 had unrestricted activities including sports and normal joint mobility, 2 had unrestricted activities with occasional pain or effusion and 1 had slight modification of his daily activities. Among 6 patients treated with resection, one with acromion resection had unrestricted activities including sports with normal strength and joint mobility, the other one with acromion resection had unrestricted activities with occasional pain and less strength in abduction, three had slight modification of their daily activities while the last one with proximal humerus resection had joint mobility limitation with restriction of daily life activities. The patient treated with ankle arthrodesis after Ewing’s sarcoma resection had a MSTS score of 23/30.

## Discussion

The incidence of local recurrence of CBL reported in literature is variable and depend on different factors. Curettage is associated with a variable rate of LR ranging from 0 to 39% [2–4, 7, 8, 12–16] with several potential risk factors reported, including age, site and secondary ABC [2–4, 11, 12, 21, 22]. In our series, the incidence of LR after intralesional curettage was 7.2%, and the only significant risk factor was represented by age < 11 years.

The presence of an open physis was reported as a risk factor because the surgeon’s fear of injuring the growth plate prevents an adequate curettage as reported by different authors [2, 8, 12].

**Fig. 5:**
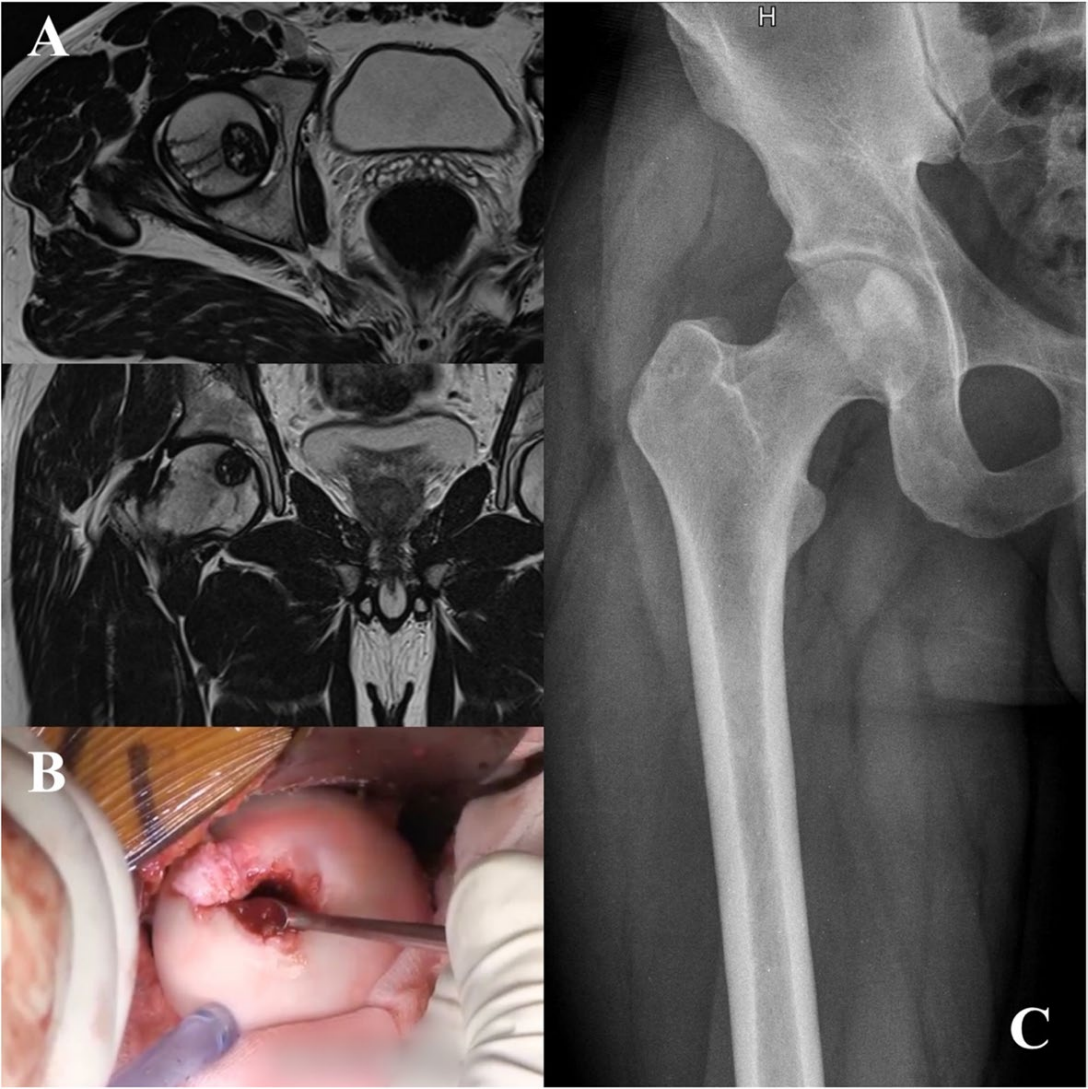
A 27 year-old patient with femoral head CBL (**A**) treated with intralesional curettage through direct anterior approach and hip dislocation (**B**) and filling with allogenic graft (**C**)

Specific sites, as femoral head, talus and posterior area of proximal tibia, can be extremely challenging [2–4]. The proximal femur is the third most common site affected by CBL after proximal tibia and proximal humerus [3], with a lower local recurrence risk in trochanteric than femoral head lesions. This could be related to the difficult surgical access at the femoral head, particularly in young patients with an open growth plate, with epiphyseal CBL very close to the physis and articular cartilage. In our series, proximal femur was one of the most affected sites in accordance with the literature [2, 3, 7, 11, 15]. Several treatment options were reported for CBL in this site. [3, 15, 23]. Lateral trans-trochanteric approach is burdened by a higher risk of LR and growth plate injury in children [3]. It requires to violate the growth plate and it was burdened by a higher recurrence rate, probably related to the difficulties in performing meticolous curettage of the femoral head. Differently, direct access to the femoral head allows excellent exposure of the lesion and careful curettage. As reported by Strong et al., direct access certainly allows a direct visualization of the entire lesion and should be performed whenever possible, although vascular injury and degenerative joint disease remain potential complications [24]. In our series no LR was observed with direct anterior approach to femoral head, in accordance with other authors [3, 7, 23] confirming the efficacy of this technique (Fig. 5). Although anterior arthrotomy pose the risk of femoral head avascular necrosis, we didn’t observe this complication, which was rarely described in literature [3, 23]. We therefore believe that using Smith-Petersen anterior approach and opening the anterior capsule with a “T” shape incision not larger than required for epiphyseal curettage, disruption of blood supply is minimized, with low risk of femoral head necrosis.

The presence of a secondary ABC is often seen in CBL and it was indicated as another risk factor for recurrence [4, 17, 25], although other series didn’t confirm these findings [2, 12].

Although our experience didn’t include minimal invasive procedures, RFA is another attractive option for small lesions localized in challenging surgical sites as femoral head, posterior aspect of proximal tibia and tarsal bones, with an incidence of LR comparable to curettage [3, 26–28]. Due to the learning curve, authors observed a higher incidence of recurrence in the early stage of application of this method, but they concluded RFA can be a successful treatment option of CBL in all anatomical sites [3, 26–28].

In locally aggressive lesions, with extensive epiphyseal involvement and articular surface disruption, segmental resection and reconstruction can be indicated with the aim of tumor removal restoring a functional joint [4]. In our series, six patients were treated with resection with a mean MSTS score of 27.4 and one of them (16.6%) developed LR 128 months after primary surgery. Acromion is a rare site of occurrence of CBL with a high risk of recurrence after curettage due to the anatomical features [29–31].

Growth disorders can be expected after aggressive curettage of CBL in patients with open physis, reported in literature with a frequency ranging from 7 to 100% [2, 8–10]. Xiong et al. [9] observed a shortened limb, ranging from 1.5 to 30 mm, in all of 18 patients with open epiphyseal growth plate treated for CBL with curettage and alcohol as local adjuvant. Huang et al. [8] reported early closure of the physis in five patients (11.9%) due to physeal damage, requiring osteotomy correction in two of them. In our series, at last follow-up early closure of the physis was observed in five patients (7.2%) causing a shortening of the affected limb ranging from 0.5 to 2 cm, in one case associated with a genu valgum. The site involved was proximal humerus, proximal and distal femur, proximal and distal tibia. All of these patients had a CBL with invasion of the growth plate; none required corrective surgery and limb shortening was managed with an orthosis. No premature closure of the physis was observed in 16 patients with open physis after curettage and local adjuvants. Thus, we believe that growth plate injuries were caused by tumor destruction rather than surgery.

Benign lung metastasis exceptionally occur but only a few series reported them, with an incidence ranging between 0.4% and 3.3% [3, 5, 11, 32]. In our series, so as so in others [2, 4, 7, 12], no distant metastasis were detected at last follow-up. On contrary, we assessed the onset of an Ewing’s Sarcoma in the same site of a previous active distal tibial CBL treated with curettage ad augmentation with cryotherapy and phenol 29 months after index surgery. Hystological specimens were reviewed by well-trained pathologist in Musculo-skeletal pathology, confirming the diagnosis of CBL. This could be explained as a rare case of malignancy development after curettage and grafting for a benign lesion, as already reported by other authors [33].

Considering CBL an epiphyseal tumor affecting primarily children and young adults, long term functional result and complications as osteonecrosis and osteoarthritis should be considered in treatment decision making. After treatment with aggressive curettage, some authors showed high incidence of secondary osteoarthritis. Farfalli et al. [7], in a series of 53 patients treated with curettage, reported a local recurrence rate of 8% and an incidence of osteoarthritis of 38% at a mean follow-up of 78 months (24 to 213), focusing on the priority of local control over the risk of secondary osteoarthritis. On the other hand, Liu et al. [14] reported only one case (2.8%) of mild osteoarthritis and one LR in a series of 36 patients, treated with aggressive curettage for CBL around the knee. Several factors have been related with secondary osteoarthritis: epiphyseal site of the lesion, iatrogenic damage curettage-related of articular cartilage, blood supply impairment after direct approach to the femoral head, and possible necrosis induced using adjuvants as liquid nitrogen or phenol [7]. We assessed secondary osteoarthritis of the adjacent joint in 12 patients (17.4%) treated with curettage. In accordance with Farfalli et al. [7], we observed most frequently progression to osteoarthritis in patients with talar and femoral head CBL. Probably this finding could be explained by the peculiar blood supply of these areas: the disruption of retinacular vessels of the hip and arteries of the tarsal canal during surgical approach might contribute to the evolution in osteoarthritis [34]. In our series, the rate of secondary osteoarthritis was lower than reported by Farfalli et al. [7]. This is probably related to the low number of patients in our series with talar and femoral head CBL. Liu et al. [14] reported a low rate of osteoarthritis (2.8%), analyzing only CBL around the knee. In our series the incidence of osteoarthritis was 17.7% in proximal tibia, 14.3% in distal femur and 50% in patella tumors. The higher incidence of osteoarthritis around the knee in our series can be conditioned by different follow up and the use of local adjuvants [2, 11, 18, 35]. Nevertheless, in our series, eight patients with arthritis were asymptomatic, three had mild symptoms without limitation of daily living activities, and only one patient with CBL of the femoral head developed severe osteoarthritis, requiring total hip arthroplasty 13 years after primary surgery. Moreover, arthritis in lower limb was more frequent than in upper limb regardless of subchondral bone involvement, due to higher mechanical stresses weight bearing related.

Our study has several limitations. The first is the inclusion of different anatomical sites and the relative small number of cases, but CBL is a rare lesion and only a few series in the literature focus on a single location with specific sites described only as single case reports. Another limitation is the inclusion of patients with open, closing and closed growth plate, with consequent possible bias on the assessment of long-term complications such as deformity, aseptic necrosis and osteoarthritis. The third limitation is different surgical treatment with a few resections, in selected cases, and most of cases treated with aggressive curettage with different local adjuvants.

## Conclusions

Aggressive curettage is the mainstay of treatment for CBL. However, complications as growth plate damage, aseptic epiphyseal necrosis and osteoarthritis are possible. Some locations such as the femoral head and the posterior side of tibial epiphysis are more difficult to treat and can be burdened by a higher incidence of recurrence and complications. Resection of CBL should be considered in case of subtotal epiphyseal extension with articular surface disruption and when the curettage would not guarantee a possible functional reconstruction.

## Data Availability

The datasets used and/or analysed during the current study are available from the corresponding author on reasonable request.

## Abbreviations

CBL: Chondroblastoma
APC: Allograft-prosthesis composites
LR: Local recurrence
ABC: Aneurismal bone cyst
RFA: Radiofrequency ablation
MSTS: Musculoskeletal Tumor Society
