# Correction: Long term outcome of surgical treatment of chondroblastoma: analysis of local control and growth plate/articular cartilage related complications

**DOI:** 10.1186/s12891-023-06328-7

**Published:** 2023-03-23

**Authors:** Francesco Muratori, Roberto Scanferla, Giuliana Roselli, Filippo Frenos, Domenico Andrea Campanacci

**Affiliations:** 1 Department of Orthopaedic Oncology and Reconstructive Surgery, AOU Careggi, Florence, Italy; 2 Department of Radiology, AOU Careggi, Florence, Italy

**Correction: BMC Musculoskelet Disord 24, 139 (2023)**

10.1186/s12891-023-06239-7

Following publication of the original article [1], the authors identified an error in the names of all authors. The given name and family name were reversed.

**The incorrect author names are:**

Muratori Francesco

Scanferla Roberto

Roselli Giuliana

Frenos Filippo

Campanacci Domenico Andrea

**The correct author names are:**

Francesco Muratori

Roberto Scanferla

Giuliana Roselli

Filippo Frenos

Domenico Andrea Campanacci

The author group has been updated above and the original article [1] has been corrected.
